# Supplementary material for: Inequalities in childhood pneumococcal conjugate vaccine uptake in England before and after the change from a 2 + 1 to 1 + 1 schedule: a longitudinal study
Source: Lancet Reg Health Eur. 2026 Apr 1;65:101667. doi: 10.1016/j.lanepe.2026.101667 (PMC13185937; doi:10.1016/j.lanepe.2026.101667)
Supplement: Text S1 to S6 [file mmc1.pdf]

Supplementary Material

Inequalities in childhood pneumococcal conjugate vaccine uptake in England before and after the change from a 2+1 to 1+1 schedule: a longitudinal study

Praise Ilechukwu MSc[1], Daniel Hungerford, PhD[2,3,4], Neil French, Phd FRCP[2], Edward M. Hill, PhD[3,4,5,6,*]

[1]Department of Health Data Science, University of Liverpool, Liverpool, United Kingdom.

[2] The Centre for Global Vaccine Research, Institute of Infection, Veterinary and Ecological Sciences, University of Liverpool, Liverpool, United Kingdom.

[3]Civic Health Innovation Labs, University of Liverpool, Liverpool, United Kingdom.

[4]NIHR Health Protection Research Unit in Emerging and Zoonotic Infections, University of Liverpool, Liverpool, United Kingdom.

[5]Department of Public Health Policy and Systems, Institute of Population Health, University of Liverpool, Liverpool, United Kingdom.

[6]The Pandemic Institute, Liverpool, United Kingdom.

*Corresponding author: Edward M Hill (Edward.Hill@liverpool.ac.uk)

Table of Contents

S1 Text. COVER data preparation 2

S2 Text. COVER data cleaning 3

S3 Text. UTLA stratification by IMD quintile 4

S4 Text. Cumulative VT IPD susceptibility estimates indicate widening socioeconomic inequalities 6

S5 Text. Additional PCV uptake figures 7

S6 Text. Findings robust to alternate vaccine effectiveness assumptions 10

References 13

S1 Text. COVER data preparation

We downloaded all pneumococcal vaccine uptake data from the COVER programme for the years 2013 to 2024, spanning a total of 52 quarterly files. These files varied in structure, format (.xlsx, .ods), and naming conventions. To ensure consistency, we renamed all files using a uniform format: 20XX_QX for pre-2020 files and 20XX QX for post-2020 files.

We imported all quarterly PCV uptake files into R and combined them into a single dataset for analysis. Due to inconsistencies across years—including changes in sheet names, column headers, and data formatting—we wrote custom cleaning scripts to extract only the relevant PCV tables (i.e., 12-month and 24-month PCV coverage and population denominators). We filtered out unrelated tables (e.g. MMR, Hib/MenC), and harmonised all extracted columns using a consistent naming convention (e.g., PCV_12m, PCV_24m, ONS_Code). [Table S1]

To prevent data type mismatches, we initially read all columns as character vectors, before converting them to appropriate types for analysis (e.g., numeric for coverage percentages and denominators, factor for categorical variables). To ensure that we handled missing values consistently, we recoded any value starting with 'N' (e.g., "N.A.") or containing extraneous symbols (such as those beginning with '[' e.g [z]) as NA.

Table S1. Variables used for data preparation.

Variables	Meaning
ONS_Code	ONS Upper Tier Local Authority code (geographic unit of analysis)
PCV_12m	Percentage uptake of the pneumococcal vaccine at 12 months
PCV_24m	Percentage uptake of the booster dose (at 24 months)
Population_12m	Eligible population denominator for 12-month coverage
Population_24m	Eligible population denominator for 24-month coverage
Year	Year the data relates to (e.g. "2019/2020")
Quarter	Quarter of the data year (e.g. Q1, Q2, Q3, Q4)
Timepoint	0 = Q1 2013, 1 = Q2 2013, etc.
Vaccine_Schedule	Binary indicator: 0 = 2+1 schedule (pre-2020), 1 = 1+1 schedule (2020+)

NB: As documented on the COVER data, there are a variety of reasons why some entries in the original data files are censored, including but not limited to:

a. Small denominators, with data censored to protect the identities of patients
b. Data quality issues
c. Data missing or unavailable
d. Unrecognisable code from EMIS: These data are collected through routine reporting by Child Health Information Systems (local NHS digital systems that track childhood immunizations and health records, including EMIS - Egton

Medical Information Systems, which is one of the main GP IT systems used across England; for more details, visit https://digital.nhs.uk/services/gp-it-futures-systems/im1-pairing-integration/emis-pfs-suppliers).

S2 Text. COVER data cleaning

Cross-referencing quarterly COVER files against the 2019 Index of Multiple Deprivation UTLA list identified two authorities that did not appear in all years: Bournemouth, Christchurch and Poole (E06000058) and Northamptonshire (E10000021).

- **Bournemouth, Christchurch and Poole (E06000058):** This authority code first appeared in the COVER dataset from 2019/2020 onwards. It was absent from COVER data for 2013/2014 through 2018/2019, where instead there were two separate authorities: Bournemouth (E06000028) and Poole (E06000029).
- **Northamptonshire (E10000021):** This authority code appeared in COVER data from 2013/2014 through 2020/2021. It was absent from the dataset from 2021/2022 onwards. During this same period, two new authority codes appeared: West Northamptonshire (E06000061) and North Northamptonshire (E06000062).

We also identified two authorities whose ONS code changed during the study period: Buckinghamshire and Dorset.

- **Buckinghamshire:** We verified ONS code consistency across all years and standardised records to the current code (E06000060).
- **Dorset:** Early COVER files used code E10000009, while later files (2020 onwards) used code E06000059 for the same geographic area. We standardised all records to use E06000059 to ensure temporal consistency.

These temporal variations resulted in 149 distinct UTLA codes appearing across the study period, though not all 149 appeared in every quarterly file. The final analytical dataset comprised 7,003 observations with no records excluded due to missing or invalid geographic identifiers.

S3 Text. UTLA stratification by IMD quintile

We merged our cleaned vaccine uptake dataset with the IMD dataset using the ONS code as a key. We retained only matched records. We then split IMD scores into quintiles using the ntile() function, with quintile 1 corresponding to the least deprived and quintile 5 to the most deprived local authorities.

We list below the UTLA's that were classified under each IMD quintile. These classifications used UTLA-level Index of Multiple Deprivation (IMD) summary data from the English Indices of Deprivation 2019 [1]. For each IMD quintile we list the UTLA's alphabetically.

IMD quintile 1 (Least Deprived): Bath and North East Somerset, Bracknell Forest, Bromley, Buckinghamshire, Cambridgeshire, Central Bedfordshire, Cheshire East, Dorset, East Riding of Yorkshire, Gloucestershire, Hampshire, Harrow, Hertfordshire, Kingston upon Thames, Leicestershire, Merton, North Yorkshire, Oxfordshire, Richmond upon Thames, Rutland, South Gloucestershire, Surrey, Sutton, Warwickshire, West Berkshire, West Sussex, Wiltshire, Windsor and Maidenhead, Wokingham, York.

IMD quintile 2: Barnet, Bedford, Bexley, (Bournemouth, Christchurch and Poole*), Camden, Cheshire West and Chester, Derbyshire, Devon, Dorset, East Sussex, Essex, Havering, County of Herefordshire, Hillingdon, Kent, Milton Keynes, North Somerset, Northamptonshire^, Nottinghamshire, Reading, Redbridge, Shropshire, Solihull, Somerset, Staffordshire, Suffolk, Swindon, Trafford, Wandsworth, Warrington, Worcestershire.

IMD quintile 3: Brent, Brighton and Hove, Bury, Cornwall, Coventry, Croydon, Cumbria, Dudley, Ealing, Greenwich, Hammersmith and Fulham, Hounslow, Isle of Wight, Kensington and Chelsea, Kirklees, Lambeth, Lancashire, Lincolnshire, Medway, Norfolk, North Lincolnshire, North Tyneside, Northumberland, Slough, Southend-on-Sea, Stockport, Telford and Wrekin, Thurrock, Waltham Forest, Westminster.

IMD quintile 4: Barnsley, City of Bristol, Calderdale, County Durham, Darlington, Derby, Enfield, Gateshead, Haringey, Islington, Leeds, Lewisham, Luton, Newcastle upon Tyne, Newham, Peterborough, Plymouth, Portsmouth, Redcar and Cleveland, Rotherham, Sefton, Sheffield, Southampton, Southwark, Stockton-on-Tees, Torbay, Tower Hamlets, Wakefield, Wigan, Wirral.

IMD quintile 5 (Most Deprived): Barking and Dagenham, Birmingham, Blackburn with Darwen, Blackpool, Bolton, Bradford, Doncaster, Hackney, Halton, Hartlepool, City of Kingston upon Hull, Knowsley, Leicester, Liverpool, Manchester, Middlesbrough, North East Lincolnshire, Nottingham, Oldham, Rochdale, Salford, Sandwell, South Tyneside, St. Helens, Stoke-on-Trent, Sunderland, Tameside, Walsall, Wolverhampton.

* For analysis of data from 2013 to 2019, we applied the IMD quintile assignment for 'Bournemouth, Christchurch and Poole' to 'Bournemouth' and 'Poole' (i.e. IMD quintile 2)

^ For analysis of data from 2021 to 2025, we applied the IMD quintile assignment for 'Northamptonshire to 'North Northamptonshire' and 'West Northamptonshire' (i.e. IMD quintile 2).

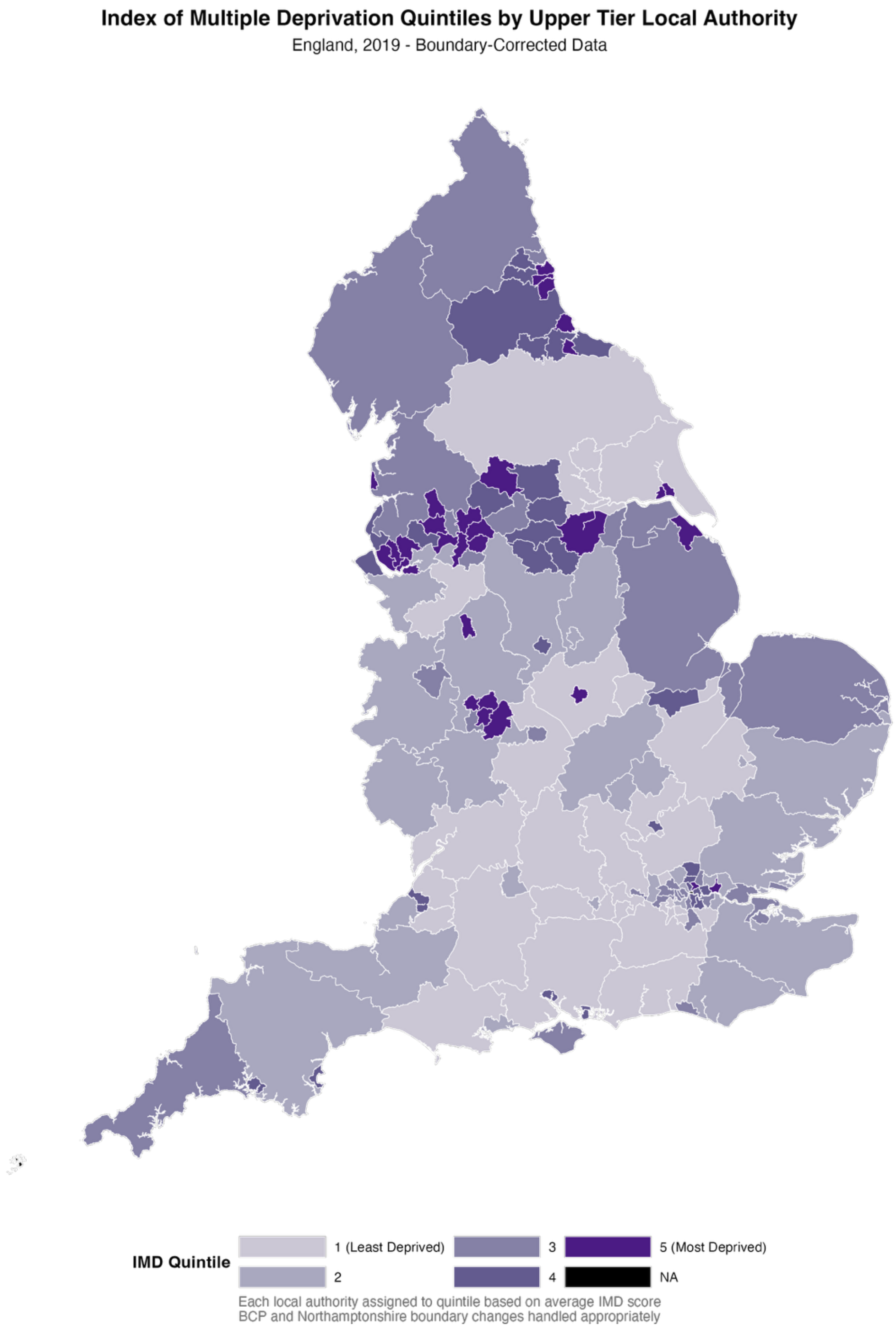

Figure S1. Assignment of UTLAs in England to IMD quintiles. *These classifications used UTLA-level Index of Multiple Deprivation (IMD) summary data from the English Indices of Deprivation 2019* [1]*. We assigned each UTLA to an IMD quintile based on the average IMD score for each UTLA. Shading denotes the IMD quintile associated with that UTLA, with the lightest shading representing the least deprived UTLAs (quintile 1) and the darkest shading representing the most deprived UTLAs (quintile 5). We assigned as NA the UTLAs not retained for our study (Isle of Scilly, The City of London).*

S4 Text. Cumulative VT IPD susceptibility estimates indicate widening socioeconomic inequalities

To better understand the growing burden of disease risk over time, we calculated the cumulative number of susceptible children by birth cohort (quarterly). This analysis demonstrates how even small reductions in uptake can lead to a steady accumulation of susceptible individuals, which may have implications for herd protection and future disease resurgence if left unaddressed [Figure S5a]. Susceptibility patterns revealed persistent and widening inequalities by deprivation level. Although overall susceptibility levels appeared similar before and after the schedule change, the more deprived areas (quintiles 3 and 5) experienced disproportionately higher vulnerability throughout the study period [Figure S5b].

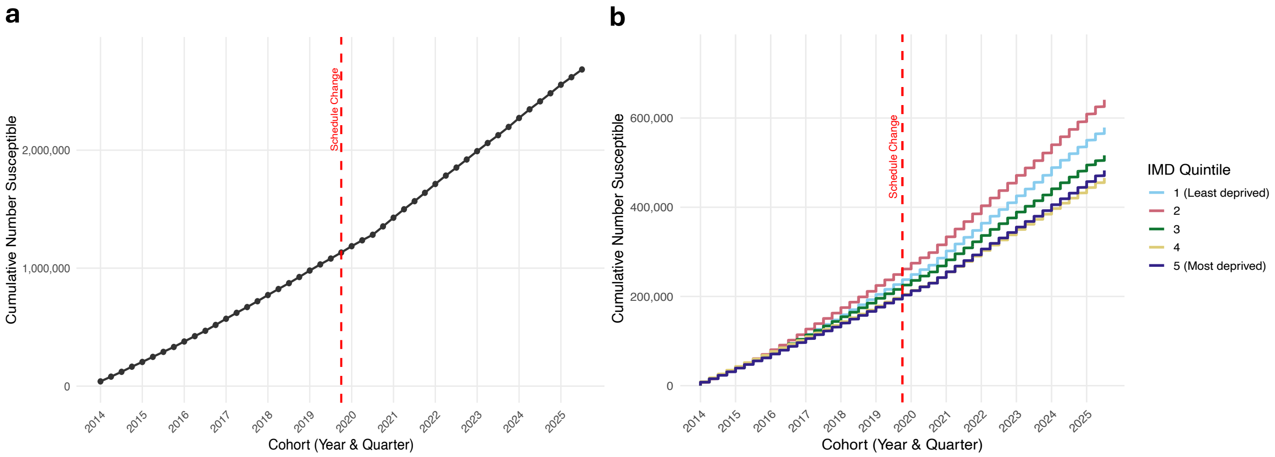

Figure S2. Cumulative number of susceptible children by quarter. *We calculated susceptibility on observed uptake and literature-based vaccine effectiveness.* ***(a)*** *Each point represents the total number of children estimated to remain susceptible to invasive pneumococcal disease (IPD) after vaccination, cumulatively summed from 2013/2014 Q2 to 2024/2025 Q3.* ***(b)*** *Stratification by deprivation quintiles. (1 = least deprived, 5 = most deprived). The x-axis tracks cohort entry by year and quarter.*

S5 Text. Additional PCV uptake figures

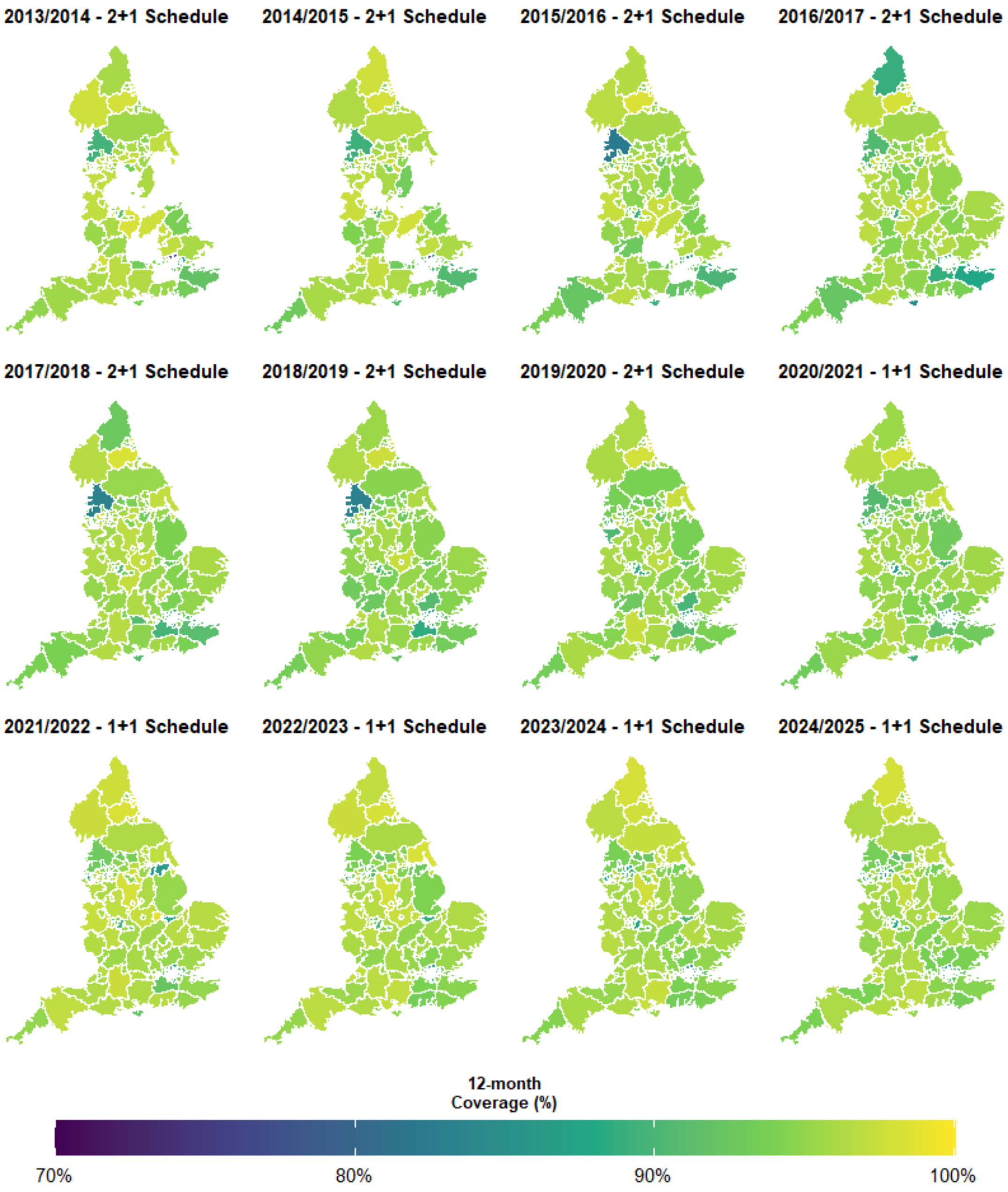

Figure S3. Geographic variation in PCV primary coverage (12 months) across local authorities in England, 2013/2014 to 2024/2025. *The colour scale ranges from dark blue/purple (70% coverage) to bright green/yellow (100% coverage), so brighter areas indicate higher vaccination rates while darker areas show lower uptake. The maps are arranged chronologically from left to right, top to bottom, spanning both the 2+1 schedule period (2013-2020), and the 1+1 schedule period (2021-2025).*

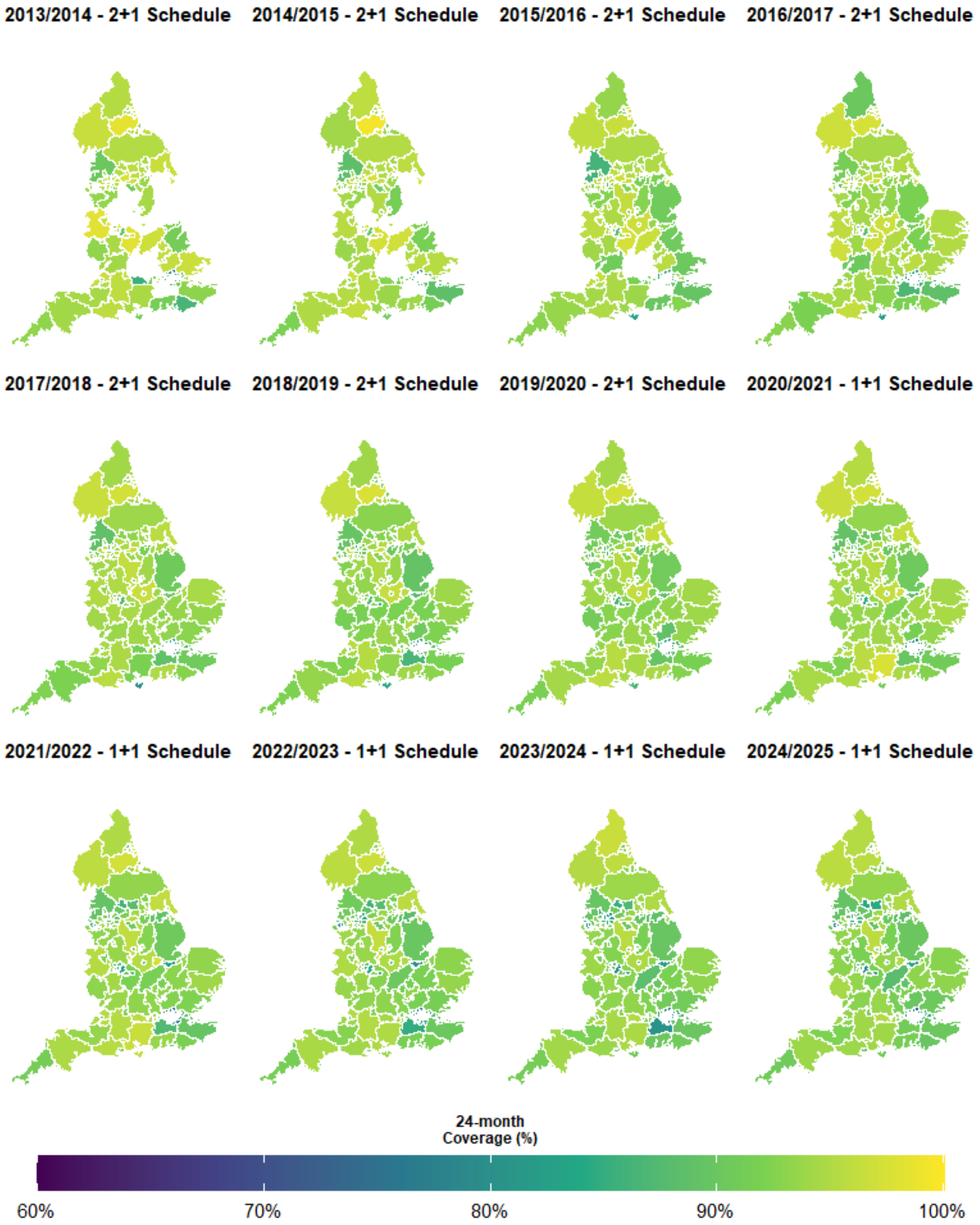

Figure S4. Geographic variation in PCV booster coverage (24 months) across local authorities in England, 2013/2014 to 2024/2025. *The colour scale ranges from dark blue/purple (60% coverage) to bright yellow (100% coverage), where brighter green/yellow areas indicate higher booster uptake and darker blue areas show lower coverage. The maps are arranged chronologically from left to right, top to bottom, spanning both the 2+1 schedule period (2013-2020), and the 1+1 schedule period (2021-2025).*

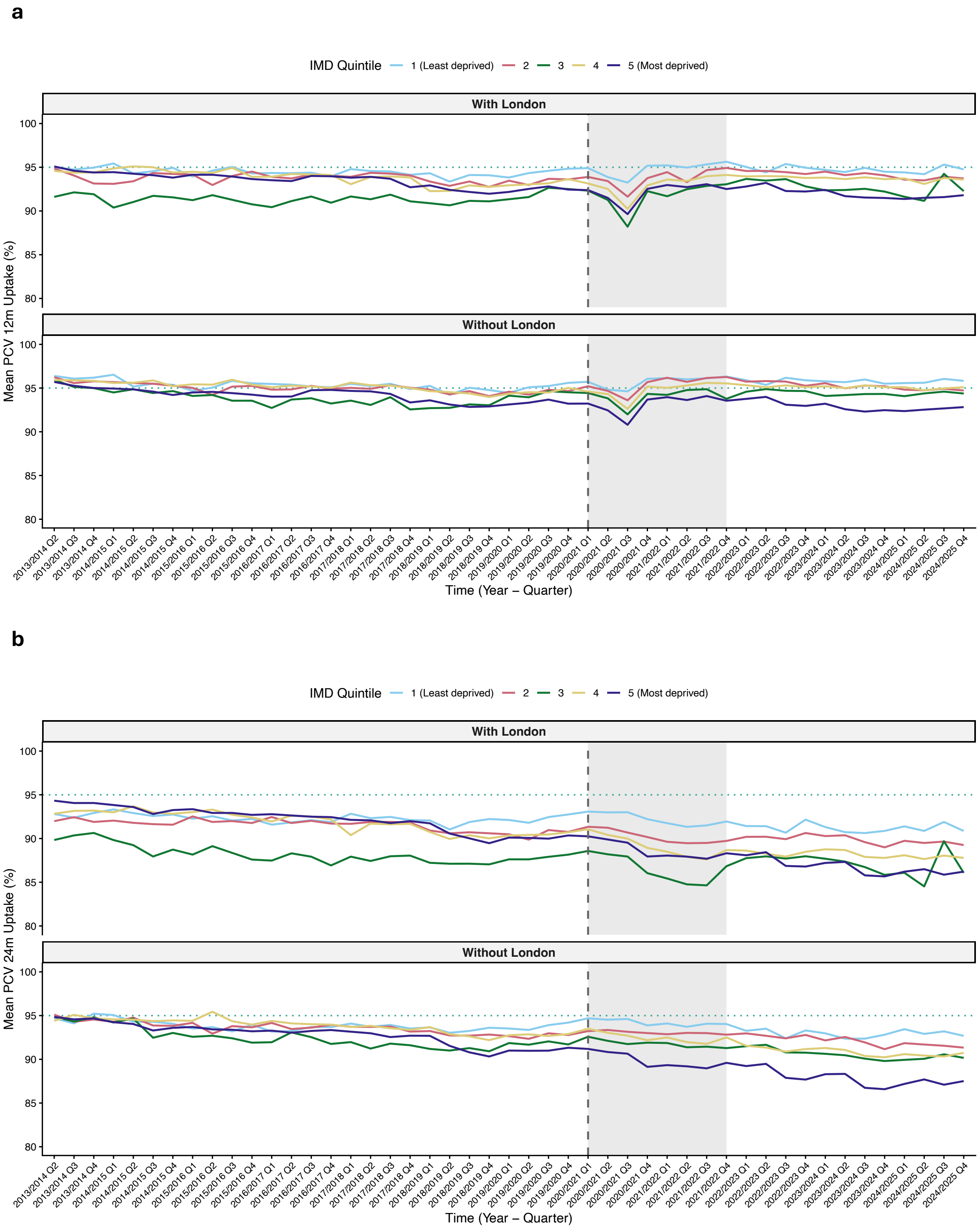

Figure S5. Impact of London on PCV Coverage by deprivation quintile. *Comparison of uptake trends for:* ***(a)*** *primary doses at 12 months;* ***(b)*** *booster dose at 24 months. In each panel, the upper plot includes 32 London boroughs (i.e. all London boroughs except the City of London), whereas the lower panel does not include any London boroughs. Lines represent IMD quintiles (1 = least deprived, 5 = most deprived). Dashed vertical line: schedule change (January 2020); shaded area: COVID-19 period. The impact of London exclusion is more pronounced for booster coverage than primary doses.*

S6 Text. Findings robust to alternate vaccine effectiveness assumptions

When applying the alternate central vaccine effectiveness assumption in our susceptibility calculation, throughout most of the study period, we found quintiles 3 and 5 (most deprived) had the highest susceptibility levels, while quintile 1 (least deprived) generally maintained the lowest susceptibility [Figure S6]. Geographic patterns remained consistent across both VE assumptions, with similar spatial distributions of susceptibility levels and no dramatic changes in regional vulnerability patterns [Figure S7].

When applying either the lower or upper vaccine effectiveness estimates, rather than the central vaccine effectiveness estimates [Table 1], we found qualitative patterns were maintained [Figures S8 & S9]. Quantitatively, comparing susceptibility estimates to when applying the central vaccine effectiveness assumption (where susceptibility estimates across IMD quintiles were between 25-31%), the susceptibility estimates were elevated when applying the lower vaccine effectiveness assumption (between 42-48%; Figure S8) and reduced when applying the higher vaccine effectiveness assumption (between 15-22%; Figure S9).

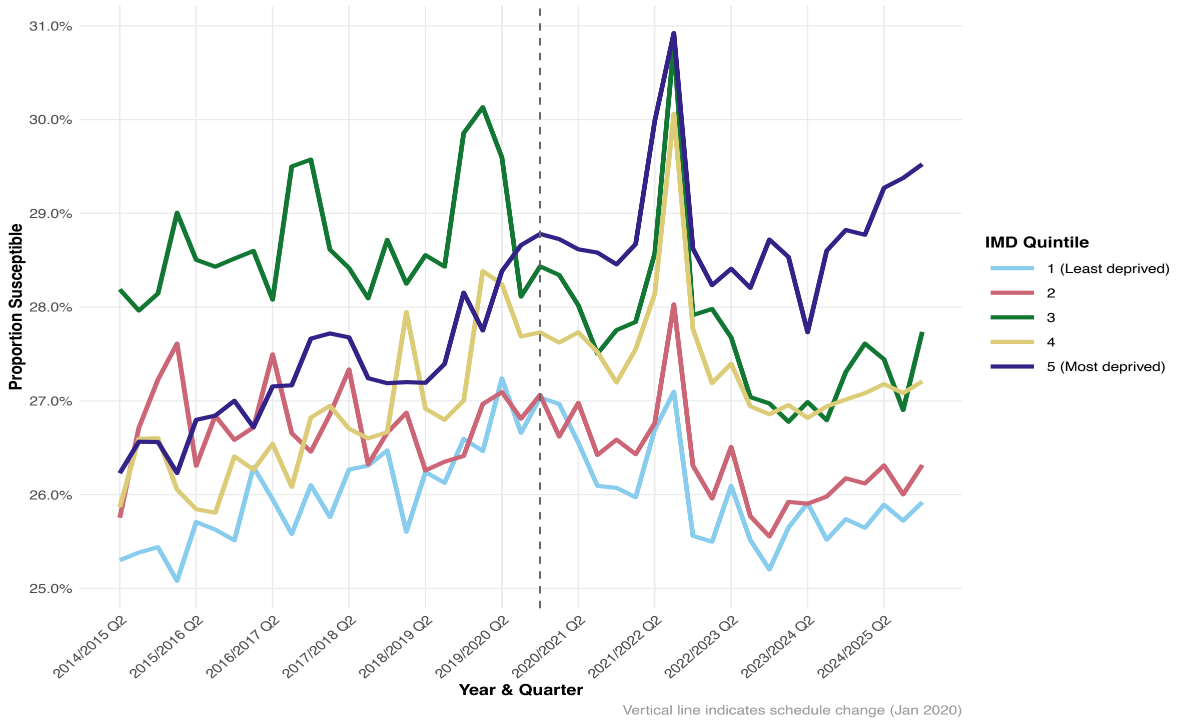

Figure S6. Estimated susceptibility to invasive pneumococcal disease by deprivation quintile: Alternate central vaccine effectiveness assumption (1+1 primary dose VE = 76.1%). Lines represent IMD quintiles (1 = least deprived, 5 = most deprived). The vertical dashed line marks the January 2020 schedule change. We observe persistent deprivation gradients and elevated vulnerability in quintiles 3 and 5 throughout the study period.

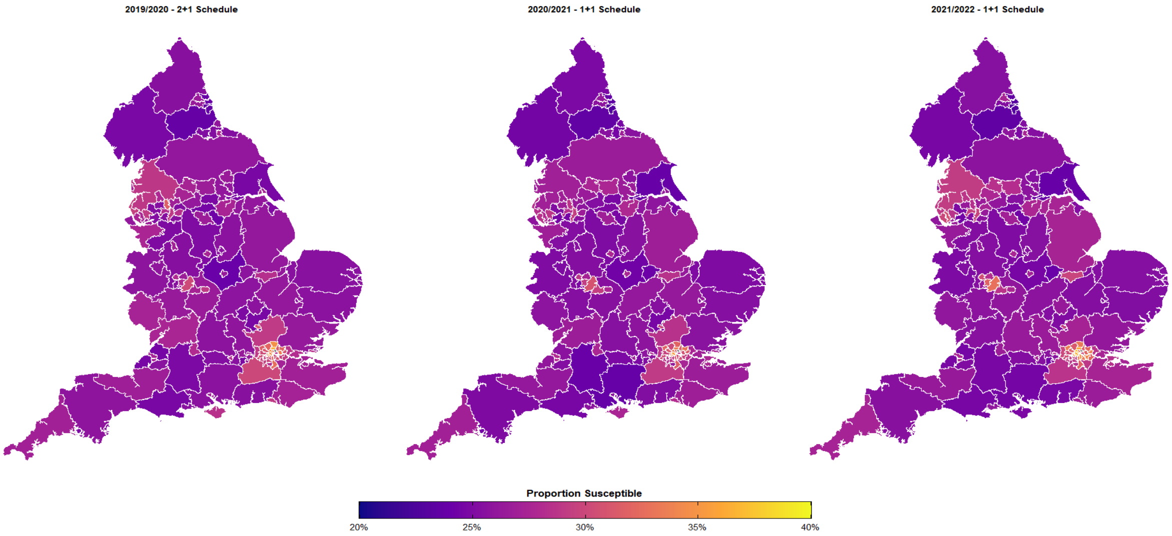

Figure S7. Geographic variation in IPD susceptibility: Alternate central vaccine effectiveness assumption. *We show average susceptibility by upper-tier local authority for 2019/2020 (2+1 schedule), 2020/2021 (transition), and 2021/2022 (1+1 schedule). Colour scale: purple (20% susceptibility) to yellow (35% susceptibility). Estimates based on alternate vaccine effectiveness assumption (1+1 primary VE = 76.1%, matching completed 2+1 primary course with booster 78.2%). Most areas show 20-30% susceptibility, with persistent pockets of higher vulnerability in urban centres.*

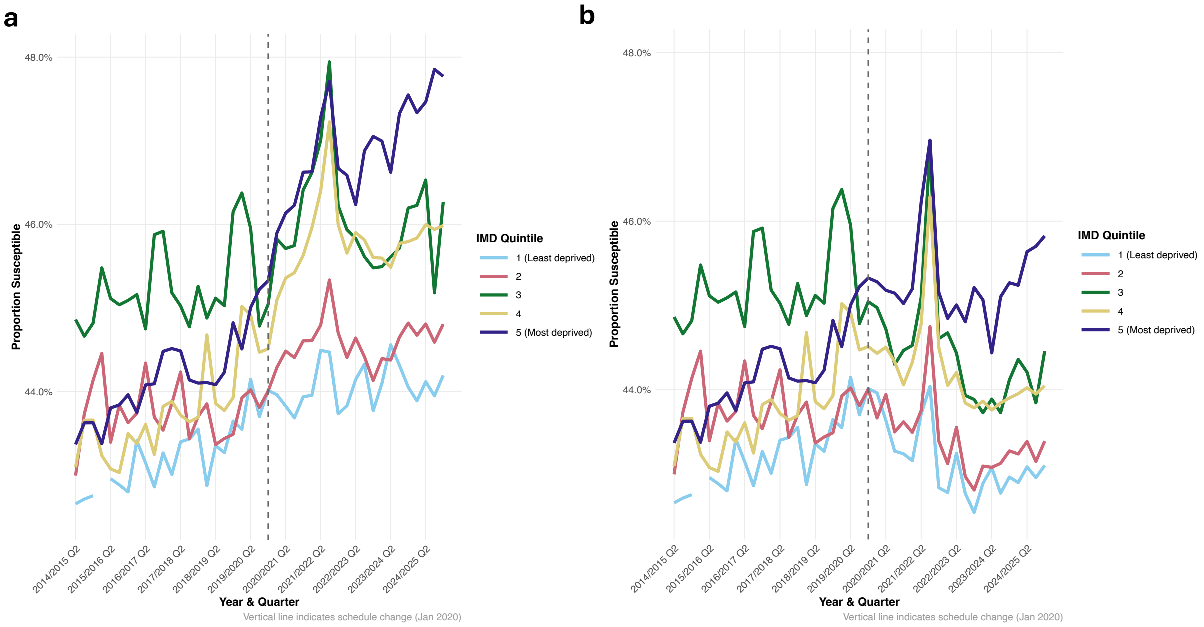

Figure S8. Estimated susceptibility to invasive pneumococcal disease by deprivation quintile: Baseline vs Alternate lower vaccine effectiveness assumptions. **(a)** Baseline lower vaccine *effectiveness assumption. **(b)** Alternate lower vaccine effectiveness assumption. Both scenarios show qualitative similar patterns to the estimates for the central vaccine effectiveness assumptions. Quantitatively, for both the baseline and alternate scenarios the susceptibility estimates are elevated when applying the lower vaccine effectiveness assumption compared to when applying the central vaccine effectiveness assumption.*

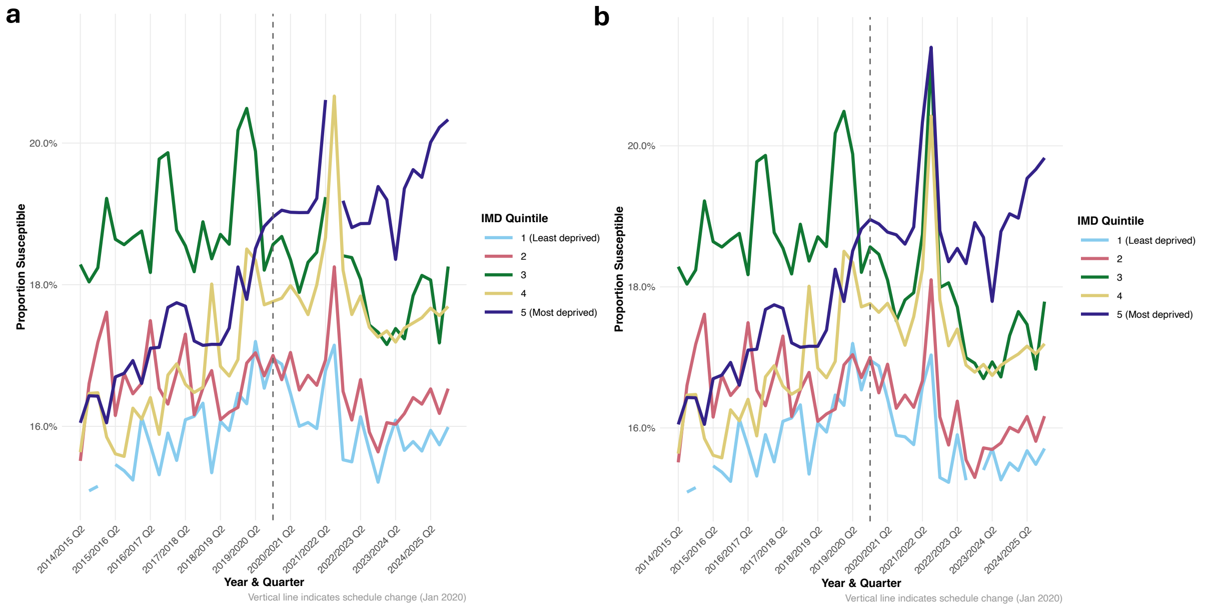

Figure S9. Estimated susceptibility to invasive pneumococcal disease by deprivation quintile: Baseline vs Alternate upper vaccine effectiveness assumptions. **(a)** Baseline upper vaccine *effectiveness assumption. **(b)** Alternate upper vaccine effectiveness assumption. Both scenarios show qualitative similar patterns to the estimates for the central vaccine effectiveness assumptions. Quantitatively, for both the baseline and alternate scenarios the susceptibility estimates are reduced when applying the lower vaccine effectiveness assumption compared to when applying the central vaccine effectiveness assumption.*

References

1 Ministry of Housing, Communities & Local Government. English indices of deprivation 2019. GOV.UK. 2019. https://www.gov.uk/government/statistics/english-indices-of-deprivation-2019 (accessed Jan 30, 2026).
